# Factors facilitating and hindering South Asian immigrant adults from engaging in exercise and physical activity – a qualitative systematic review

**DOI:** 10.1186/s12889-024-18288-1

**Published:** 2024-05-18

**Authors:** Nasimah Maricar, Behram Khan, Trixy David, Kimme L Hyrich, Anne Barton

**Affiliations:** 1 Northern Care Alliance NHS Foundation Trust, Salford Royal NHS Foundation Trust, Manchester, UK; 2 Centre for Epidemiology Versus Arthritis, Centre for Musculoskeletal Research, Division of Musculoskeletal and Dermatological Sciences, University of Manchester, Manchester, UK; 3 Department of Health Professions, Manchester Metropolitan University, Manchester, UK; 4 The Kellgren Centre for Rheumatology, Manchester University NHS Foundation Trust, Manchester, UK; 5 NIHR Manchester Biomedical Research Centre, Manchester Foundation NHS Trust, Manchester Academic Health Science Centre, Manchester, UK; 6 Centre for Genetics and Genomics Versus Arthritis, Centre for Musculoskeletal Research, Division of Musculoskeletal and Dermatological Sciences, University of Manchester, Manchester, UK

**Keywords:** Exercise, Physical activity, South Asian, Qualitative, Systematic review

## Abstract

**Background:**

Exercise and physical activity are key components of management in patients with rheumatic musculoskeletal diseases (RMD), but people of the South Asian communities have a lower level of engagement with these activities compared to their Caucasian counterparts. The aim of this qualitative systematic review was to determine the barriers and facilitators of exercise and physical activity in South Asian communities who have migrated and live in western countries, particularly in those who have RMD.

**Methods:**

Qualitative studies, published in English between 1999 and 2021 and including evaluation of barriers and/or facilitators to exercise or physical activity behaviour in people of South Asian adult communities who have migrated and/or lived in western countries were identified from Embase, MEDLINE, CINAHL, PsycINFO, Google Scholar and manual searches. The studies were appraised using the CASP checklist. Inductive thematic synthesis was used to identify common and global themes.

**Results:**

A total of 32 studies that discussed barriers and facilitators of physical activity in South Asian communities who have migrated and lived in western countries were used for this review but there were no studies identified that focussed specifically on those with RMD. Following appraisal of the reporting of the studies, 30 studies were included in the pooling of the results. The facilitators and barriers to physical activities were broadly categorized into ‘extrinsic’ and ‘intrinsic’ factors. Extrinsic factors such as ‘opportunity’ included environmental factors such as weather and safety; socioeconomic factors such as education, language and literacy, and support in the form of social, psychological and resources. Intrinsic factors included cultural factors, such as life stages and family influence, beliefs and knowledge, which impacted attitudes and skills.

**Conclusions:**

This review has synthesised evidence of barriers or facilitators and identified potentially modifiable factors influencing physical activity and exercise engagement, which could form the basis of evidence-based interventions to promote participation in healthy behaviour change. Provision of a safe, comfortable and culturally acceptable environment together with culturally-aligned cognitive strategies to facilitate acquisition of exercise-efficacy skills could help engagement.

**Registration:**

The systematic review was registered on PROSPERO, registration no. 289,235.

**Supplementary Information:**

The online version contains supplementary material available at 10.1186/s12889-024-18288-1.

## Background

The benefits of exercise and physical activity (EPA) are well-established and appear to be one of the most important interventions that can prevent, treat and alleviate chronic non-communicable diseases [1–6]. The use of EPA in the management of joint pain is widely advocated [7] and exercise as a treatment has been recommended in international guidelines for rheumatic musculoskeletal diseases (RMD) [8–11]. People with RMD have chronic pain, which may mean that people with RMD could avoid activity known to cause pain. However, inactivity increases their joint stiffness and weakens muscles, exacerbating their pain and making performing of daily activities more difficult. Some people are also fearful of doing exercises as these could lead to pain or discomfort. However, exercises are important in maintaining movements in joints and to strengthen the muscles that surround and support the joints. In turn, that can improve pain or allow more activity before pain arises. Although chronic pain may be persistent, the pain level or intensity and frequency can be reduced through modifying activities, such as maintaining a normal body weight. Exercise and engaging in physical activities are important tenets for chronic pain management and a wide range of evidence-based exercise interventions such as walking, tai chi, strengthening, neuromuscular training and aquatic exercise, among others, have been shown to effectively improve pain and function for RMD [8–11]. The European Alliance of Associations for Rheumatology (EULAR) has proposed a list of general and disease-specific barriers for engagement with physical activity and facilitators that can aid people with RMD to be more physically active. However, these are knowledge-based and condition-related factors and there is an absence of focus on community or cultural influences [11].

There is a distinction between physical activity and exercise with the former defined as “any bodily movement produced by skeletal muscles that results in energy expenditure,” whereas exercise is a subset of physical activity defined as a “planned, structured, and repetitive bodily movement done to improve or maintain physical fitness” (p. 129) [12]. Physical activity among South Asian (SA) communities, however, is reported to be significantly lower than their Caucasian counterparts [13–18]. SA people, belonging to or having ancestors from India, Pakistan, Bangladesh, Nepal, Bhutan, Sri Lanka or Maldives, are also identified as a high-risk group for developing early onset of metabolic syndrome, that includes cardiovascular disease, hypertension, type 2 diabetes mellitus, and dyslipidemia [19, 20]. A recent systematic review investigating objective measures of physical activity and sedentary behaviour among SA adults, found that only 53–61.1% met physical activity guidelines of 150 min/week of moderate to vigorous physical activity [18]. Furthermore, the perception of SA women that they are not sedentary during the day is not supported by objectively assessed results, with around 45–55 min of each hour spent being sedentary [21]. In a United Kingdom (UK) study where types and levels of physical activity were measured using a multidimensional index, Indians, Pakistanis and Bangladeshis were reported to undertake statistically significantly less physical activity than those of European ancestry [16].

Three previous systematic reviews have investigated physical activity engagement and barriers to it among SA communities living overseas [13, 22, 23]. In one systematic review of qualitative studies [22], barriers and facilitators of physical activity uptake and adherence among older SA from Canada and UK were identified and categorized into four key themes: communication that encompasses language barriers; relationships as in support network; beliefs of ill health consequential to ageing and fate, and environmental factors such as weather, safety, accessibility and acceptability of exercise facilities. In another systematic review investigating physical activity among SA women, a lack of understanding of the recommended amounts of physical activity and its benefits as well as cultural and structural barriers to physical activity were identified, while faith and education could serve as facilitators [13]. One other systematic review investigated generational differences in the physical activity of UK SA and suggested second-generation SA might have a more favourable attitude towards physical activity than the first-generation [24]. However, although second-generation SA may be more physically active than the first-generation, they were still less active than the White British [24]. That review highlighted significant socio-economic and cultural heterogeneity among UK Indians, Pakistanis and Bangladeshis, and hence the same approaches to increasing physical activity might not be appropriate for all people of these ethnic groups [24]. The importance of tailoring interventions to address population-specific barriers was reinforced by Koshoedo [23], which included studies of SA and African-Caribbean people and, whilst some common themes relating to the barriers to physical activity emerged, there were also important differences influenced by migration history, such as whether they were first generation of migrants; experiences and exposure to EPA including prior education and lifestyle; culture-related preferences for single gender classes or modest attires, and health beliefs, including fatalism.

Several studies have identified lack of fluency in English as a barrier to participation and attendance in physical activity programmes [25–27]. This may be compounded by these populations not being approached or effectively engaged in the efforts offered by health providers to improve their health [27–30]; for example, in an Australian study evaluating referrals by general practitioners to exercise physiologists for the management of chronic diseases where physical activity formed part of the standard component of clinical care, patients from non-English-speaking backgrounds were referred at less than half the rate (0.41 per 1000) of those from English speaking backgrounds (0.96 per 1000) [31].

Despite the presence of a number of past systematic reviews, no significant milestone has been achieved in improving the engagement of EPA in the SA communities in the last decade. Whilst there may be an awareness among some SA people of the need to undertake EPA, many have not put this lifestyle advice into practice. To address health inequalities, there is a paramount need to better understand the issues surrounding EPA engagement in individuals from SA communities who have migrated to western countries. The aim of the current systematic review is to improve our understanding of the issues surrounding the engagement of EPA among SA communities including factors that may facilitate or hinder their engagement through appraising the literature on the barriers, challenges and facilitators of EPA in SA communities, and including findings from studies that have been published since the last systematic review [23] in order to identify (i) common findings across studies; (ii) studies with a specific focus on SA with RMD, and (iii) gaps in knowledge and where further research is needed. The findings could serve as the foundation for informing tailored treatment interventions effectively for SA patients with RMD.

## Methods

The systematic review was registered on PROSPERO (registration no. 289,235) in 2021. Results from eligible articles were qualitatively synthesised using the framework synthesis approach and reported according to the Enhancing Transparency in Reporting the Synthesis of Qualitative Research (ENTREQ) statement [32].

### Theoretical framework underpinning the work

An inductive thematic synthesis was undertaken, broadly on the basis of the thematic analysis for synthesizing qualitative studies described by Thomas and Harden [33]. This includes a process of translating concepts or themes from one study to another. A process of thematic networking was used to map and link themes into basic, organizing and global themes [34].

### Eligibility criteria

Publications were selected for the review if they met the following criteria:(i) published in the English language;(ii) focus on SA adults who have migrated and/or lived in western countries; and (iii) discuss barriers or enablers to exercise or physical activity behaviours. We included studies from 1990 until end of October 2021. Only qualitative studies (involving interviews in group or individually) were eligible for inclusion; for studies that contained both quantitative and qualitative methodologies, only the qualitative element of the study was used for this review.

### Information sources/Search strategy

The search strategy included an exploration of publications from electronic databases such as Embase, MEDLINE, CINAHL, PsycINFO and Google Scholar as well as manual literature searches including searching the reference lists of included studies. The databases were searched individually for all possible terms and combination of terms to accommodate differences in their search engines. All medical subject-heading searches (MeSH) were exploded where possible. The search terms ‘physical activity’, ‘exercise’, ‘barriers’, ‘enablers/facilitators’, ‘cultural’, ‘religious’, ‘ethnic groups’, ‘ethnicity’ and ‘South Asian’ were used. The search terms related to South Asian ethnicity were, ‘Asian’, ‘Indian’, ‘Pakistani’, ‘Bangladeshi’ and any other South Asian ethnic groups available. Specifically, the terms ‘physical activity’ or ‘exercise’ were combined with each of the other terms listed above.

Two series of electronic searches were conducted by two members of the team. The first was done with the help of the librarian in 2020 and the second was conducted in 2021 by another member of the team who was not involved in the initial literature search (BK). The electronic searches were repeated to ensure inclusion of new studies that had been published since the last literature search and to ensure the literature search was robust.

### Study selection

From the initial search strategy, publications were excluded if they did not meet the inclusion criteria, as determined by the title and abstract. This was done by two members of the team (NM, BK) where the identification process and screening were performed independently. Subsequently, full text articles of potentially relevant publications were read and those meeting the eligibility criteria were included including publications that looked at SA and Caucasian communities where the inclusion of the latter provided an insightful comparison. The scores to the Critical Appraisal Skills Programme (CASP) checklist [35] were also used to exclude studies; any study with 4 or more missing and/or unclear items were excluded.

### Risk of bias assessment

The evaluation of the reporting of the included studies was appraised using the CASP checklist for the qualitative research appraisal tool [35]. Two different reviewers (NM, BK) scored each study independently.

### Data collection process

For studies that contained both quantitative and qualitative methodologies, only the qualitative element of the studies was used for the synthesis of this review. Relevant texts under the headings “results /discussion/conclusions” were manually extracted using data extraction sheets. Extraction of relevant information was conducted independently by two members of the team (NM, TD) where themes were manually inputted and organized. The data collected by one reviewer was checked by the other with consensus agreement should there be cases of disagreements between the two reviewers [36].

### Data items

Themes reported by the authors of each study were extracted and listed (using the authors’ original wording) as a separate heading. Findings from individual studies were then used to populate the list.

### Data synthesis

A process of reciprocal translation was undertaken, whereby each study was scrutinized for evidence of all themes arising manually. We appraised each using the original themes given in their studies. Through an interpretive thematic approach that involved the iterative process of coding and constant comparison, the description and wording of the themes were continually revised, and notes made as to how themes related and whether they could be merged [34]. The common themes of all studies were then pooled. For instance, those about weather from all studies were populated together. The process was repeated with all the other themes. Initial thematic networks were drawn to facilitate understanding of the themes, with similar themes organized and grouped together. Broad organizing themes were then identified [34]. Each organizing theme was written up descriptively, and four global themes were identified.

## Results

**Fig. 1:**
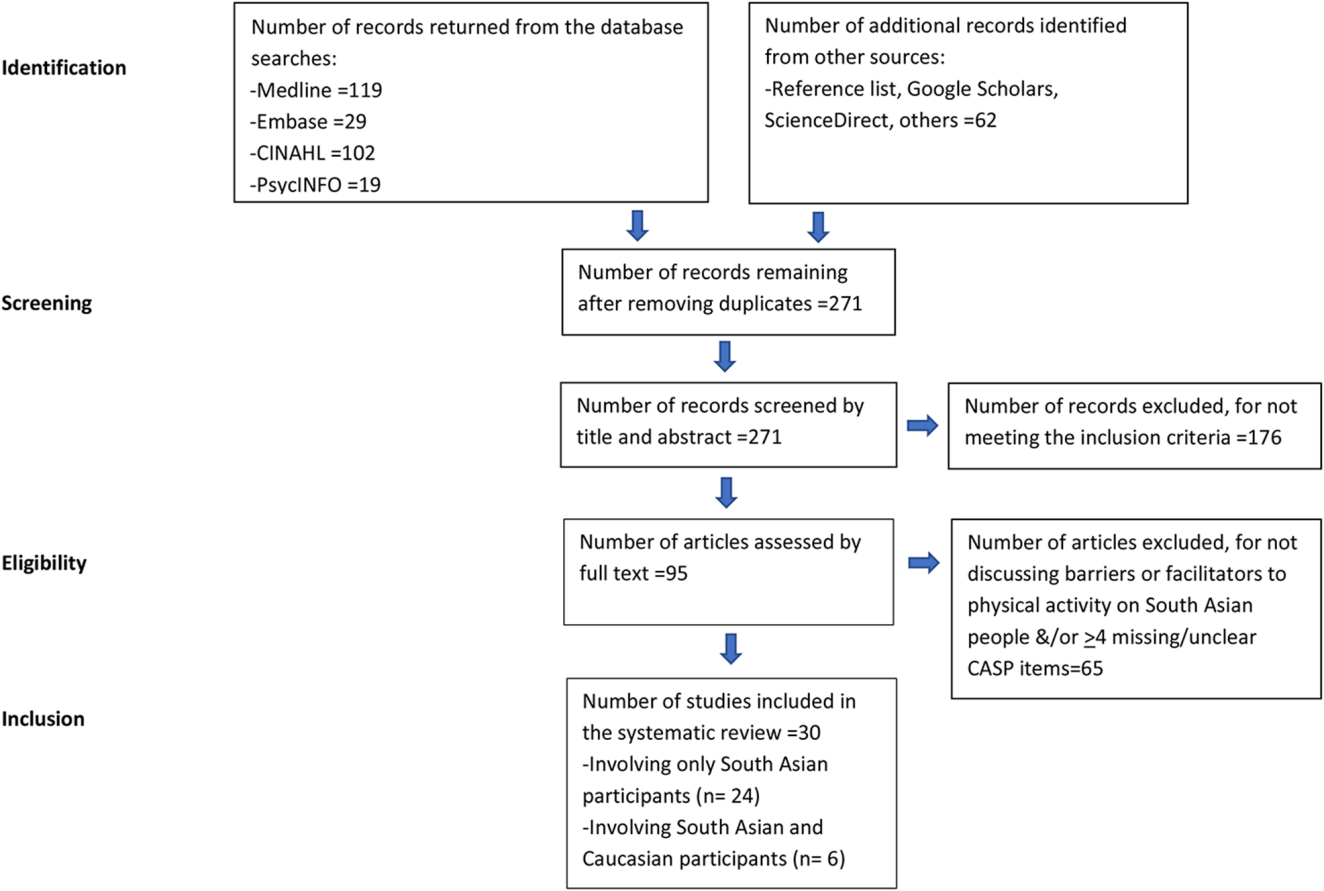
Prisma diagram

From the 330 publications screened, 30 publications were identified as meeting the inclusion criteria (Table 1) and included 6 publications that studied both SA and Caucasian communities [25, 37–41] (Fig. 1–prisma diagram). There was no study specifically focused on SA people with rheumatic and musculoskeletal diseases. Four studies included both quantitative and qualitative methodologies [21, 42–44]. No disagreement between the two reviewers was found for the data collection process and in the themes that emerged.

**Table 1:** Qualitative studies on barriers or facilitators to exercise or physical activity behaviours in South Asian adults

Authors	Setting	Design	Sample	Languages	Age (years)	Primary aims	Secondary aims
Abdulwasi et al. 2018	Ontario, Canada	12 individual, face-to-face, semi-structured interviews at their homes or mosque averaged 26 min (two interviews < 9 min)	12 South Asian Muslim women	English, Hindi, Urdu	Mean 53 Range = 23.0–74.0	Exploring decisions to participate in mosque-based physical activity intervention	
*Astin et al. 2008	West Yorkshire, UK	interviews at their homes or places of their choice lasting 60-90 min	65 men and women (20 Pakistani-Muslim; 12 Indian-Sikh; 13 Indian-Hindu; 20 White-European 54 carers (19 Pakistani-Muslim; 8 Indian-Sikh; 10 Indian-Hindu; 17 White-European)	English, Urdu, Hindi, Punjabi, Sylheti, Gujarati	Mean = 62 Range = 40–83	Exploring experiences of South Asian and White-European patients and their carers’ on the impact of coronary heart disease on them and their experience with cardiac rehabilitation	To explore exercise regimes
^∞^Bronson 2017	East London, UK	semi-structured in-depth interviews 4–6 weeks after completion of Pain Management Programme	10 Bengali women	Sylheti	-	To better understand the role of self -management in British Pain Society guidelines for group chronic pain programmes as delivered within Bengali community	To apply concepts learned for future provision of more culturally informed programs
Cross-Bardell et al. 2015	East Midland, UK	16 one-to-one semi-structured and 6 family group interviews at their homes 1 focus group discussion with local providers and stakeholders inner city Derby and Nottingham	34 men and women (17 Pakistani, 13 Indian, 4 Bengali/other South Asian) 18 relatives (16 non-English speakers) 11 health professionals	English, Punjabi, Urdu	Mean = 41 Range = 19–67	To explore perspectives on enhancing physical activity and diet among South Asians in urban deprived communities at high risk of chronic diseases	To inform development of culturally appropriate health promotion intervention
^Curry et al. 2015	Cardiff, Wales, UK	mixed-methods study with 24 semi-structured interviews out of 140 women who wore an accelerometer for 7 days	24 Bangladeshi and Pakistani women (19 fluent in English)	English, Sylheti, Bangla, Urdu, Punjabi	Mean = 52.8 SD = 10.1	To compare perceived physical activity and sedentary time to objectively measured data	To explore physical activity- and sedentary time-specific contexts, experiences, and sources of physical activity and sedentary time
Daniel et al. 2018	US	1 focus group	40 Indian women (25 Hindu, 14 Sikh, 1 Christian)	English, Hindi	Mean = 50 Median = 50.6 SD = 7.0	To examine perspectives of South Asian Indian immigrant women to barriers to and motives for lifestyle physical activity	
*Darr et al. 2008	West Yorkshire, UK	in-depth semi-structured interviews	65 patients (20 Pakistani-Muslim, 13 Indian-Hindu, 12 Indian-Sikh, and 20 Europeans)	Urdu, Hindi, Punjabi, Sylheti, Gujarati, English	Pakistani-Muslim, mean 59, range 46–72; Indian-Sikh, mean 63, range 48–79; Indian-Hindu, mean 63, range 40–82; European, mean 66, range 42–83	To examine and compare illness beliefs of South Asian and European patients admitted within the previous year with unstable angina or myocardial infarction, or to undergo coronary artery bypass surgery about causal attributions and lifestyle change	To explore perception of physical activity and exercise
Dave et al. 2015	Chicago, Illinois, US	6 focus groups	42 Asian Indian and Pakistani women	Hindi, Urdu, English	Mean = 42	To elicit definitions of and experiences with exercise, facilitators and barriers, benefits and costs, and their ideas about potential interventions	
Farooqi et al. 2000	Leicester, UK	6 focus groups each lasting between 40 min to 1 h	44 South Asian men and women attending community centres	Hindi, Gujarati Punjabi	Mean = 55	To identify key issues relating to knowledge of an attitude tool lifestyle risk factors for coronary heart disease	To explore perception of physical activity and exercise
Galdas et al. 2012	British Columbia, Canada	15 face-to-face semi-structured interviews lasting about 1 h at their homes	15 Punjabi Sikh men and women	Punjabi, English	Mean = 63	To describe Punjabi Sikh patients who were attending cardiac rehabilitation education program on their perceived barriers to engaging in physical exercise following myocardial infarction	
Grace et al. 2008	London, UK	17 focus groups and 8 semi-structured interviews	80 Bangladeshi men and women, 29 Islamic scholars and religious leaders, 20 health professionals	Sylheti, English	Mean = 35 SD = 2 Mean = 35 SD = 8 Mean = 41 SD = 8	To explore the attitudes, values, and beliefs of first and second generation Bangladeshi participants without diabetes towards the prevention of type 2 diabetes	To explore perception of physical activity and exercise
*Gupta et al. 2017	Australia	semi-structured interviews from 45 min to > 2 h	57 men and women (41 South Asian and 16 Anglo-Australian)	English, Hindi	Mean = 60	To compare perceptions, barriers, and facilitators of physical activity in these groups who had type 2 diabetes and/or cardiovascular disease	
*Horne et al. 2009	North West England, UK	15 focus groups 40 individual semi-structured interviews	87 men and women (29 South Asian, 58 Caucasian) 40 men and women (17 South Asian, 23 Caucasian)	English, South Asian languages	Mean = 65.7 Mean = 64.8	To identify salient beliefs that influence uptake and adherence to exercise for fall prevention among community dwelling Caucasian and South Asian 60–70 year olds	
*Horne et al. 2010	North West England, UK	15 focus groups lasting between 1–2 h 40 individual semi-structured interviews lasting 30-90 min	87 men and women (29 South Asian, 58 White) 40 men and women (17 South Asian, 23 White)	English, Punjabi, Gujarati	Mean = 66.1 Mean = 65.0	To explore the influence of primary health care professionals in increasing exercise and physical activity among 60–70-year-old White and South Asian community dwellers	
Horne et al. 2012	North West England, UK	focus groups In-depth interviews	29 Indian and Pakistani men and women 17 Indian and Pakistani men and women	English, Punjabi, Gujarati	Mean = 66.1 Mean = 65.2	To identify attitudes and beliefs associated with the uptake and adherence of physical activity among community-dwelling South Asians aged 60–70 years	
*Horne et al. 2013		focus groups in-depth Interviews 60–90 min	87 men and women (58 European, 7 Indian, 22 Pakistani) 40 men and women (23 European, 6 Indian, 11 Pakistani)	English, Gujarati, Punjabi, Urdu	Mean = 65.6 Mean = 65.0	To explore the barriers to initiating and maintaining regular physical activity among UK Indian, Pakistani and White British adults in their 60s	
Jepson et al. 2012	Aberdeen, Glasgow and Edinburgh, UK	9 focus groups and 10 in-depth semi-structured interviews	59 Bangladeshi-, Indian- and Pakistani-origin men and women	English, Punjabi	-	To explore motivating and facilitating factors likely to increase physical activity for South Asian adults and their families, in order to develop successful interventions and services	
^∞^Johnson 2000	England	22 group discussions (14 with South Asian groups) Survey	Indian, Pakistani, Bangladeshi	-	-	To report behaviours including inter-ethnic differences in beliefs, perceptions and barriers to physical activity and exercises in South Asian people	
Kalra et al. 2004	Northern California, US	8 focus groups	57 Asian Indians women and men	English, Punjabi	-	To gather information on the perceptions of cardiovascular risk within the Asian Indian community, and to identify opportunities to design health promotion and intervention programs for Asian Indian communities	To explore perception of physical activity and exercise
^Kalavar et al. 2004	US	questionnaires (Study 1) focus groups (Study 2)	100 Asian Indians 10 Asian Indians	English English, Hindi/ Gujarati	Mean = 73.0 SD = 5.0 Mean = 71.9 Range = 66–79	To investigate the motives for (Study 1) and barriers to (Study 2) participation in physical activity by older Asian Indian immigrants to US	
^Khanam et al. 2008	Borough of Tower Hamlets, East London, UK	Interviews lasting 20–30 min in gym private room	25 British Bangladeshi women	English, Sylheti	Mean = 47.4 SD = 9.1	To investigate the attitudes and beliefs held by UK Bangladeshi women on health and exercise and explore possible ways of increasing levels of physical activity in this group	
Lawton et al. 2006	Edinburgh, UK	interviews averaged 1 h in their homes usually	32 (9 Indians,23 Pakistani)	Punjabi, Urdu, Hindi, English	-	To explore Pakistani and Indian patients’ perceptions and experiences of undertaking physical activity as part of their diabetes care	
Mohan et al. 2008	Sydney, Australia	semi-structured, in-depth interviews in their homes	13 Asian Indians (8 patients and 5 family members)	English	-	To report lifestyle factors of Asian Indians in Australia in relation to coronary heart disease and to identify factors that could inform health education and rehabilitation programs for migrant Asian Indians in Australia	To explore perception of physical activity and exercise among a series of lifestyle factors
Netto et al. 2007	Edinburgh, UK	2 focus groups 6 months apart	91 Bangladeshi, Indian and Pakistani men and women	Hindi, Urdu, Punjabi, Sylheti, English	-	To explore how service user perspectives can be used to develop effective, culturally focused chronic heart disease prevention interventions by addressing identified barriers, including deeply held cultural beliefs	To explore perception of physical activity and exercise among a series of beliefs and lifestyle factors
Ollife et al. 2009	British Columbi, Canada	Participants’ observations and 36 semi-structured individual interviews averaged 60 min	36 South Asian men	Punjabi, English	Mean = 69.5 SD = 8.3	To better understand how masculinity informs and influences men’s physical activity	
Penn et al. 2008	Breckon Hill, Abingdon and Green Lane, UK	9 group interviews in community venues where they attended their physical activity sessions, lasting between 60 and 75 min	20 Pakistani females	English	Mean = 33.5	To investigate Pakistani female who had Type 2 diabetes on their perspectives of behaviour change and of salient intervention features	
Rathnanaswami et al. 2016	Canada	a semistructured focus group, 3 individual and 1 in-depth follow-up interview	8 South Asian women (4 Sri Lankan, 2 Pakistani, 2 Bangladeshi)	English	Mean = 45.57	To understand physical activity experiences of South Asian women employees working at a South Asian Women’s Centre in Canada and their perceptions of new SA women immigrants with regards to barriers and facilitators to EPA participation	
Riley et al. 2013	New York, US	6 focus groups	67 Bangladeshis	Bangla	Mean = 42	To understand factors that affect physical activity practices and weight management in this community	
Sriskantharajah et al. 2007	Birmingham, UK	one-to-one, semi-structured interviews in their homes, lasting 1 to 2 h	15 South Asian women (5 Indian, 4 Pakistani,1 Bangladeshi, 2 East African Asian, 3 Sri Lankan	English, South Asian languages	Mean = 52	To explore influences on, and attitudes towards, physical activity among South Asian women with chronic heart disease and diabetes to inform secondary prevention strategies	
Victor 2014	England, UK	109 interviews lasting about an hour (range 25–100 min)	109 men and women (50 Bangladeshi and 59 Pakistani)	Urdu, Sylheti	-	To explore how people aged 50 + from Bangladeshi and Pakistani communities talk about physical activity in terms of their daily lives	
^Williams et al. 1999	Manchester, UK	13 semi-structured interviews	13 Asian women	English, Asian languages	-	To follow up overweight/obese Asian women who participated in the healthy eating and exercise group programme and examine their perceptions of the programme and changes in weight	
Yeowell 2010	North west of England, UK	6 interviews in own homes or social club	6 Pakistani Muslim female patients	English, South Asian language	-	To gain an insight into the needs of female Pakistani service users in relation to physiotherapy	

Index: UK-United Kingdom; US-United States; SD-standard deviation. *Studies involving South Asian and Caucasian; ^Studies with quantitative and qualitative data where only the latter was used for the review. ^∞^ Articles excluded due to poor CASP scores

Agreement in the studies’ reporting scores was achieved in all but five cases, which had only minor disagreements. In instances where grading differed, agreement was achieved through consensus. The summary of the CASP scores is given in Table 2. We found several studies lacked information about the relationship between the researcher/s and the participants, who the interviewers were, whether the same interviewers were used throughout the studies, and if the interviewers were part of the research team. A number of studies also had not declared if ethics approval had been sought. In some studies, there was a lack of transparency on how the data was analysed and its underpinning theory. The two studies [26, 45] with the lowest scores were deemed to have poor reporting of their studies and were excluded from the analyses and results [46].

**Table 2:** Summary of scores for appraisal of articles using CASP checklist [35]

	Clear aims	Appropriate methodology	Appropriate research design	Appropriate recruitment strategy	Data collected addressed research issue	Consideration of researcher /participants relationship	Ethical consideration	Rigorous data analyses	Clear statement of findings	Research valuable?
Abdulwasi et al. 2018	Y	Y	Y	Y	Y	Y	Y	Y	Y	Y
Astin et al. 2008	Y	Y	Y	Y	Y	Y	Y	Y	Y	Y
^∞^Bronson 2017	Y	Y	Y	Y	Not clear	Y	N	Not clear	Not clear	Y
Cross-Bardell et al. 2015	Y	Y	Y	Y	Y	Y	Y	Y	Y	Y
Curry et al. 2015	Y	Y	Y	Y	Y	Y	Y	Y	Y	Y
Daniel et al. 2018	Y	Y	Y	Y	Y	Y	Y	Y	Y	Y
Darr et al. 2008	Y	Y	Y	Y	Y	Y	Y	Y	Y	Y
Dave et al. 2015	Y	Y	Y	Y	Y	Y	Not clear	Y	Y	Y
Farooqi et al. 2000	Y	Y	Y	Y	Y	Y	Not clear	Not clear	Y	Y
Galdas et al. 2012	Y	Y	Y	Y	Y	Y	Y	Y	Y	Y
Grace et al. 2008	Y	Y	Y	Y	Y	Y	Y	Y	Y	Y
Gupta et al. 2017	Y	Y	Y	Y	Y	Y	Y	Y	Y	Y
Horne et al. 2009	Y	Y	Y	Y	Y	Not clear	Y	Y	Y	Y
Horne et al. 2010	Y	Y	Y	Y	Y	Y	Y	Y	Y	Y
Horne et al. 2012	Y	Y	Y	Y	Y	Y	Y	Y	Y	Y
Horne et al. 2013	Y	Y	Y	Y	Y	Y	Y	Y	Y	Y
Jepson et al. 2012	Y	Y	Y	Y	Y	Y	Y	Y	Y	Y
^∞^Johnson 2000	Y	Y	Y	Not clear	Not clear	Not clear	Not clear	Not clear	Not clear	Y
Kalra et al. 2004	Y	Y	Y	Y	Y	Not clear	N	Not clear	Y	Y
Kalavar et al. 2004	Y	Y	Y	Y	Y	Not clear	Y	Y	Y	Y
Khanam et al. 2008	Y	Y	Y	Y	Y	Y	Y	Not clear	Y	Y
Lawton et al. 2006	Y	Y	Y	Y	Y	Y	Y	Y	Y	Y
Mohan et al. 2008	Y	Y	Y	Y	Y	Y	Y	Y	Y	Y
Netto et al. 2007	Y	Y	Y	Y	Y	Y	Y	Y	Y	Y
Ollife et al. 2009	Y	Y	Y	Y	Y	Not clear	Y	Y	Y	Y
Penn et al. 2008	Y	Y	Y	Y	Y	Y	Y	Y	Y	Y
Rathanaswami et al. 2016	Y	Y	Y	Y	Not clear	Y	Y	Y	Not clear	Y
Riley et al. 2013	Y	Y	Y	Y	Y	Y	Y	Y	Y	Y
Sriskantharajah et al. 2007	Y	Y	Y	Y	Y	Y	Y	Y	Y	Y
Victor 2014	Y	Y	Y	Y	Y	Y	Y	Y	Y	Y
Williams et al. 1999	Y	Y	Y	Y	Y	Y	Not clear	Y	Y	Y
Yeowell 2010	Y	Y	Y	Y	Y	Y	Y	Y	Y	Y

Index: Y- yes, N- no; CASP- Critical Appraisal Skills Programme, ^∞^Excluded studies due to low rating

**Fig. 2:**
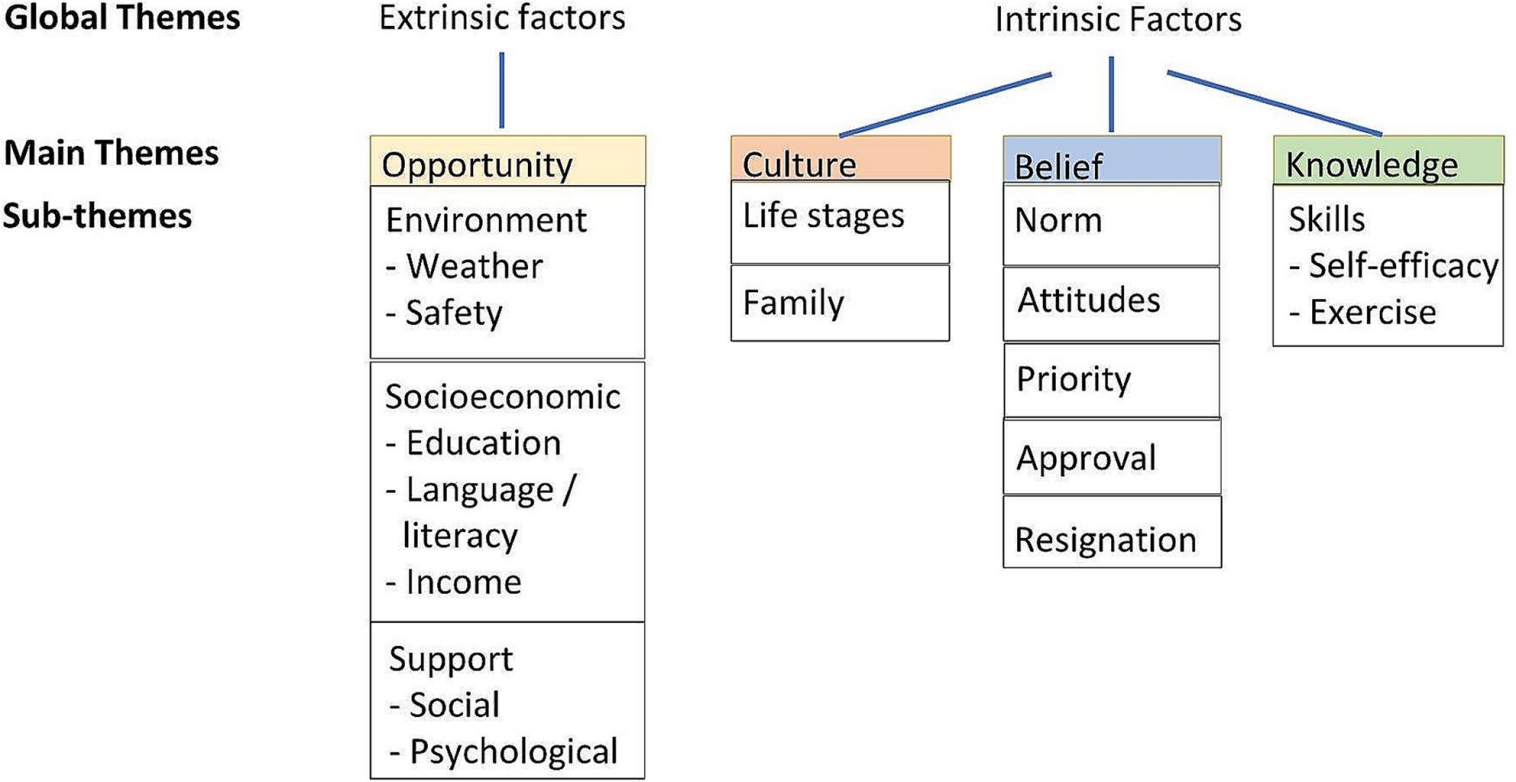
Schematic diagram of global themes, main themes and sub-themes for facilitators and barriers to physical activity engagement in South Asian communities

The global themes identified as facilitators or barriers to EPA engagement in SA communities (Table 3) could be classed as ‘extrinsic’ or ‘intrinsic’ factors (Fig. 2). Sub-themes that could promote EPA could also hinder it, that is, affecting it positively when present and negatively when compromised, or vice-versa.

**Table 3:** Barriers and facilitators to engagement of exercise and physical activity in South Asian people

Barriers	Facilitators
**OPPORTUNITY**	
**Environment**	
***Weather***	
Poor weather discourages physical activity	Good weather encourages physical activity
Changing weather affects regular physical activity	Indoor facility
Fear from suffering an injury during poor weather	Preference for physical activity in temperature-controlled environments
***Safety***	
Concern for safety	
Feeling of vulnerability and unfamiliar environment	
**Socioeconomic status**	
***Education***	
Low level of education	
***Language/Literacy***	
Low literacy level	
***Income***	
Long work hours/anti-social work hours/lack of time	
Financial constraints/poverty	
**Support**	
***Social***	
Difficulty arranging childcare to allow engaging in outdoor physical activity	Family and peer group support
	Support from health professionals
***Psychological***	
	Need a role model and others to inspire
	Encouragement/supervision from health professionals
***Resources***	
Lack access e.g. information, space, EPA	Access to free physical activity programmes
	Access to suitable physical activity programmes
**BELIEFS**	
**Norm**	
Adhering to culturally acceptable conduct	
Exercise not part of the norm	
Gender differences on activities considered as exercise	
Physical activity affected by religious festivals and practices	
Cultural acceptability of being overweight	
Constraint of modest attires	
**Attitudes**	
Preference for informal and unplanned physical activity	Awareness of health benefits of physical activity
Concern about exercise exacerbating pre-existing health problems	Awareness of the need for regular physical activity
Physiological response to exercise/physical activity viewed as illness states and to be avoided	Willingness to change into a more active lifestyle
	Motivation to exercise for weight reduction, social enjoyment e.t.c
Perceptions of ill health and injury associated with being physically active	Improved quality of life led to long-term adherence to physical activity
Anxiety and fear from unaccustomed physiological products of exercise/physical activity	Maintaining good physical and mental health through adherence to physical activity
Exercise setting deemed inappropriate	Exercise setting deemed appropriate
Presence of pain hinders willingness to exercise or perform physical activity	Second generation more positive towards exercise and physical activity
**Priority**	
Family and household responsibilities come first/guilt conscience if otherwise	
Less time dedicated for personal pursuit of physical activity	
**Approval**	
Disapproval of mixed gender environment and interaction	Religion encourages exercise and being healthy
Lack of family and peer group endorsement	
Men disapproving their female partners from going out or doing EPA	
**Resignation**	
Complacency or fatalism	
**CULTURE**	
**Life stages**	
Having younger children could lead to lack of time to be physically active	Older people may have time for exercise to help their chronic diseases
**Family**	
Caring responsibilities	Younger children can encourage outdoor play activity including walking to parks
**KNOWLEDGE**	
**Skills**	
***Self-efficacy***	
Lacking self-efficacy skills	
***Exercise***	
Poor exercise know-how knowledge	Exercise and physical activity engagement due to known health benefits e.g. increasing strength, reducing symptoms from chronic conditions
Lack knowledge on benefit of physical activity including for strength, balance, mobility	Teaching/supervision of exercise

### Extrinsic factors

‘Opportunity’ was identified as a factor impacting the ability of SA adults to engage with EPA, and this could be influenced by environment, socioeconomic status and availability of support.

Environmental factors such as weather [38, 39, 44, 47–50] could either encourage or hinder engagement with physical activity with good weather promoting engagement [39, 48], but rainy and snowy weather discouraging engagement [25, 42]. Lack of safety from physical harm and abuse decreased the likelihood of engagement with physical activity in both SA men and women [19, 25, 27, 37, 44, 47, 48, 51–53].

Socioeconomic status influenced the income and the financial viability to engage with EPA [39, 48, 54]. Educational level, language and literacy appears to affect the engagement with EPA; lack of language proficiency and low literacy levels were reported to negatively impact the ability of SA people to engage with EPA [27, 44, 48, 55, 56]. Poverty that arises from low socioeconomic status was closely associated with working long hours and having less leisure time [39, 48, 54]. Support from friends, family and health practitioners was consistently reported to facilitate positive engagement with EPA [47, 57, 58]; and the converse was true if encouragement and approval was not offered [52, 54]. Verbal encouragement and exercise supervision by health professionals were facilitators of engagement with EPA [24, 27, 53, 57]. The presence of health professionals could offer social support whilst their encouragement and supervision are likely to offer psychological support. The availability of resources such as free gym access, having a means to travel to or having a local venue for exercise classes, and community-approved exercise programmes were also important factors reported to improve physical activity level [44, 47, 57].

### Intrinsic factors

Three key intrinsic factors emerged as influencing how well an individual engages with EPA: culture, belief and knowledge; of these, culture seems to play a prominent role. For example, several studies found EPA was not ingrained within the culture or upbringing of SA communities [21, 39, 49, 54, 56, 59] and cultural conflict and acceptability was reported to discourage sport and exercise in women [52, 54, 55]. The second generation of SA who were born and brought up in western countries were reported to have more positive attitudes towards EPA [59]. Life stages and age could determine how much ‘free time’ an individual has, with older people cited as being better able to engage in EPA [59]. Family commitments in the form of responsibilities of caring for elders and children were factors discouraging engagement with EPA, especially among women [21, 49, 59]. There also appears to be cultural nuances as to what EPA may be acceptable with family activity and walking seeming to be the preferred options [59].

Engagement with EPA could also be influenced by belief systems including adhering to their norm [21, 39, 49, 54, 56, 59], prioritizing family needs [39, 44, 48, 49, 53, 59], desiring approval [48], acceptability of exercise settings [52, 54, 55] and attires [52, 54, 55, 59]. There could also be disincentive among some SA to be physically active if they believe body size is a measure of prosperity and beauty, and if being overweight was viewed favourably [43, 52, 55]. Complacency and excessive attribution to genetic predisposition could reduce the inclination to engage in EPA [38, 39, 49]. Furthermore, fear of injury or exacerbation of chronic conditions, despite the recognition of the health benefit of EPA, also acted as a barrier to engagement [25, 39, 42, 56, 60].

Knowledge also emerged as a key theme, influencing attitudes and skills acquisition. Positive attitudes towards EPA were driven by knowledge [21, 49, 58, 61]. However, lack of knowledge on what exercises to do and how to do them well, impacted confidence in engaging effectively with EPA [21, 38, 40, 41, 52, 53, 55, 56, 58, 59]. Without formal training and teaching, some felt compelled to avoid EPA so as not to suffer any negative consequences of exercise [47, 62].

Lack of self-efficacy skills, motivation and confidence affects the ability to perform exercise or to be physically active [41, 42]. This leads to individuals placing higher reliance on others and having dependency on health professional before being able to engage with EPA [41, 42, 56, 59, 63]. In contrast, having the know-how to exercise [47, 62] and empowerment [27, 37, 41, 62] including from role models [64] facilitates better engagement with EPA programmes.

### South asians compared with caucasians

When SA are compared to their Caucasian counterparts, there are distinguishable differences in the attitude towards exercise with SA being less inclined [38–40], types of exercises preferred with SA preferring walking [37, 39, 40] and also in the performance of exercise, often cited as unplanned in SA [39, 56, 60]. There are also differences in the level of teaching needed [21, 41, 53] with SA individuals requiring in-depth instruction on EPA and more detailed guidance [39]. Nonetheless, both SA and Caucasians share similar perceptions that EPA could exacerbate pre-existing health problems and result in physical harm [25, 37–41]. Sometimes their desire to exercise may conflict with perceived or self-imposed restriction that could contribute to low mood and frustration [25, 40, 56]. In both SA and Caucasians, the presence of pain can lead to reluctance to exercise [25, 42].

## Discussion

This systematic review has focused on qualitative studies that evaluated and discussed barriers and facilitators to engagement and, whilst none of the studies had a specific focus on SA patients with RMD, common themes emerged.

We found barriers and facilitators to EPA could be grouped into extrinsic and intrinsic factors. These align with the Self-Determination Theory (SDT) in conceptualising individuals’ motivation for maintaining consistent physical activity. According to Gellar et al. [65], the SDT organizes both internal and external factors driving the continuation of health behaviours. Extrinsic factors drive individuals’ physical activity to attain external or tangible rewards, while intrinsic factors are propelled by internal rewards, such as personal interest, and are anticipated to foster long-term habitual behaviours. Extrinsic factors require external issues to be addressed to enable individuals to better engage with physical activity and in our review, opportunity has been identified as a key extrinsic factor. Opportunity to engage in EPA could be facilitated in a number of different ways including, providing a safe environment [17, 53] with protection from adverse weather conditions [39, 48]; addressing socioeconomic barriers through education, improved literacy and language proficiency [27, 44, 48, 55, 56]; the presence of support, socially and psychologically [47, 57, 58] and access to relevant resources [44, 47, 57]. For SA people, language, racial harassment, dress codes, modesty and inappropriate facilities that are not gender-specific may pose further barriers [17, 27, 44, 48, 53, 55, 56, 64, 66, 67]; it may therefore be helpful if gyms and exercise venues could create a more culturally acceptable ambience through offering flexibility in their dress codes, less offensive decors and pictures, and provision of single-gender classes and changing facilities that could encourage increased participation of SA people in gym and exercise programmes [67].

Knowledge, belief and culture were three key themes identified under intrinsic factors. Inadequate knowledge appears to be on two fronts: first, the lack of awareness of the specific benefits of EPA limits engagement of SA people with it. Second, even in those individuals who are aware of the benefits of EPA, the lack of knowledge on the know-how to exercise creates fear and anxiety that prevent engagement; this includes limitation of activities to prevent physiological changes as a result of physical exertion and also out of fear of injury or exacerbating pre-existing illness and comorbidities [25, 39, 42, 56, 60]. Several papers have highlighted that reduced levels of knowledge may be linked with an external locus of control, including fate [26, 41, 42] and hinder the promotion of positive attitudes and the inculcation of self-efficacy. Whilst there are many social-cognitive health behaviour theories and frameworks available that may help to understand and target physical activity behaviour, our findings suggest that three factors may impact engagement: firstly risk appraisals, defined as one’s perceived vulnerability compared to that of others; secondly behaviour-specific outcome expectancies from EPA, and thirdly self-efficacy beliefs in the face of obstacles and barriers to adopt health behaviours [68]. Even if there is sufficient self-belief that leads to motivation to make a decision and a plan, the self-belief needs to be harnessed further into translating goals into action and then maintaining the health behaviour. In SA communities, a lack of motivation to carry through these ideas of positive change into permanent behavior change has been reported [37, 41, 62, 64]. However, knowledge acquisition is dependent on many determinants including cultural beliefs [21, 26, 39, 49, 54, 56, 59, 64, 66], the presence of social and psychological support [47, 57, 58] and access to relevant resources [44, 47, 57]. SA people appear to require further endorsement from society to enforce their desire to be active and help transform health behaviours from sedentary to being more active. They may also require additional supportive measures to sustain an active lifestyle which could be the focus of future intervention. Social norms, social support, individual motivation, environments, education, and policies, independently and in combination, are important for behavior change.

Competing responsibilities [21, 49, 59] and cultural norms [21, 26, 39, 49, 54, 56, 59] are among the most consistent factors identified as limiting EPA engagement. However, competing family demands, socioeconomic disadvantages and preference to conduct EPA in safe and socially supportive environments that provide shelter from the elements are not exclusive to people of SA [49, 51, 69–71]. In these respects, there were more similarities than dissimilarities between Caucasian and SA older adults [40, 49, 51, 69–72]. Nonetheless, there are distinct differences between Western cultures in comparison to SA cultures [38–40, 69]. In many SA societies, EPA is not yet immersed within the fabric of the societies [39, 44, 54]. The convenience of better transportation systems compared with native countries of origin may also promote sedentary lifestyles [66, 73] that could be overcome through motivation to engage in EPA as part of the new norm of living in western countries. Introducing active lifestyle early in SA people particularly girls whilst in school may also inculcate positive attitude and cultural acceptability towards EPA [67]. There are also gender-related expectations [26, 37, 43] and what are often perceived as acceptable behaviours [26, 27, 52, 56, 59] where engagement with EPA could be confused with morality or cultural identity. For example, EPA is seen as a male-related activity and that could create conflict with cultural values of SA women [26, 52, 54, 55]. Furthermore, SA societies often have strong family values where caring responsibilities supersede individual needs and aspirations and these responsibilities often fall to women [26, 39, 44, 48, 49, 53, 59]. It could also be difficult for SA women to attain self-efficacy if they are constantly needing to gain approval from others for their action [52, 54].

Education and knowledge could play a key role in overcoming barriers, including fatalism [38, 40, 49]. Language acculturation and literacy [27, 44, 48, 55, 56] including in Quranic Scriptures for those who practice Islam, probably has a part in influencing understanding as to what is permissible by their religion, and often these individuals may confuse religion opposing EPA when it is the cultural norm restricting it [64]. Highlighting Islamic teaching that advocates healthy living and importance of physical fitness can counteract fatalism and passivity [74]. There are limited studies that have reviewed the impact of other religions on exercise perception, with existing studies primarily focused on Muslims. Future studies should explore the impact of different religions and cultural milieu on exercise behaviour, and also interpersonal, intrapersonal, and environmental facilitators among the various religions and SA cultures given there is heterogeneity within the SA communities that could affect their readiness towards engaging with EPA. To facilitate successful and permanent transformation of an active lifestyle and engagement of regular EPA, future work could explore the different SA communities on their aptitude of cultural readiness towards EPA. Whilst the development of interventions was not the focus of this review, incorporating for example, Behaviour Change Wheel and COM-B framework would be invaluable when designing future intervention studies, and would result in Capability, Opportunity and Motivation considerations that could be more instructive for informing the development of interventions [75, 76].

Given that EULAR recommends exercise for the management of joint pain, it was surprising that our systematic review found that none of the studies focused on SA people with RMD. In order to tailor interventions effectively for this group of patients, it is important to understand factors that may facilitate or hinder their engagement. The review has identified modifiable factors that could form the basis of evidence-based interventions to promote participation in healthy behaviour change in this group. At a local health service level, cognitive behavioural therapy aligned to SA cultures and beliefs, and knowledge-based programmes educating on the benefit of EPA delivered in their spoken language could be offered to all SA individuals diagnosed with chronic disease including RMD [20]. This can be delivered during health care consultations or via nurse-led or physiotherapy-led clinics with the programmes extended to include family members or other support networks. Future studies may need to explore how best to achieve this including the appropriate resources, particularly for those who cannot read or write. Additionally, addressing the training and resource needs of nurses, physiotherapists, and other healthcare professionals is crucial for a successful intervention; and this needs to eventually extend to university academic curriculum training of health professionals. Gender-specific exercise classes could be offered locally, preferably led by a native speaker where possible. When this is not possible, the segregation between the genders with a partition could be acceptable so they could be shielded from the opposite gender whilst exercising. Self-referral schemes for physiotherapy could be beneficial to ensure they could get support when needed. Where social prescribing is available, relevant exercise programmes could be offered to these SA individuals. Within the community, free gym membership and local physical activity facilities could encourage further positive EPA behaviours [69]. Additionally, posters and advertisement of gym classes and exercise programmes could picture SA people exercising in their ‘culturally acceptable’ attires. The use of local SA social media platforms can help to disseminate these incentives. Further engagement with SA communities is needed including creating incentives to participate, for example by providing creche and/or childcare support while parents or grandparents are exercising. Implementing such recommendations would help promote exercise engagement regardless of ethnicity but may be particularly helpful in SA groups.

At a national level, there is a need for more concerted effort to recruit community and religious leaders to filter down positive messages about EPA to encourage health behaviour change from sedentary lifestyles into more active ones. This could involve developing partnerships with community leaders [49, 67] to create tailored health campaigns, organising community events promoting physical activity, and integrating these messages into religious gatherings or community forums. Additionally, establishing training programs or workshops for leaders on the benefits of exercise and strategies for behaviour change could enhance its effectiveness. In the longer-term we need to address socioeconomic disparities and health inequalities through social reforms and policy changes in education, housing and socioeconomy. To aid this, having SA people working within the government can add ‘voice’ and push the agenda forward.

This review has several strengths: it attempts to draw common findings from across previous studies and the various pertinent issues highlighted as barriers or facilitators to EPA. Unlike previous reviews, it includes a comparison of the common barriers and facilitators between SA and Caucasian populations, highlighting those specific to the SA community. Furthermore, it has also highlighted gaps in our understanding and identified where more work is needed including the dearth of literature on EPA in SA people with rheumatic conditions. Also this review found there was a lack of distinction made in the studies between physical activity and exercise where the lack of distinction may be less relevant in conditions such as obesity or cardiovascular diseases. However, in people with RMD joint-specific exercise is often needed. Further barriers such as language could be more an issue for exercise such as participation in a gym class with an English-speaking instructor as compared to physical activity like going for a walk in the park.

There are several limitations that should be acknowledged. First, many of the articles cover EPA as a small if not an important part of their enquiry on their risk assessment to obesity, cardiovascular system or disease, diabetes, and/or other chronic long-term illnesses. This meant that the focus on EPA might not always be the primary aspect studied, affecting the depth of analysis in their exploration of factors affecting engagement with EPA. Second, the review was limited to studies published in English; future reviews could explore literature published in other languages. Third, we considered SA as a single group but there is heterogeneity among the SA communities as they may speak different languages and may practice different religions; these could influence their engagement with EPA. Fourth, since the review was registered on PROSPERO with our search period ending in October 2021, further work has been published [77–81], but was not formally included in order to comply with the registration. However, it is important to note that the findings from those studies did not materially affect the themes identified in the current work; neither are any of these studies conducted on SA with RMD. Finally, there are inherent constraints of qualitative studies in their lack of generalisability, specificity to particular contexts, times and participant groups, as well as the inherent subjectivity in interpretation. Within qualitative analysis methodologies, there are also complexities in synthesis and the variability in methodological guidance, which poses further challenges. The synthesis of the qualitative data presented here relied on the themes identified in the studies included and we did not attempt to re-categorise according to behavioural theory, which is a theory of learning that postulates that behaviours are learned through different types of conditioning, influenced through environmental interactions. Whilst that would be an alternative way of classifying themes, the evidence synthesised in the current review identified potentially modifiable themes that could inform clinical interventions to enhance engagement. Despite these limitations, qualitative research offers an understanding of intricate human behaviours, beliefs, and social phenomena. It provides rich and in-depth insights that quantitative methods might not capture. Mixed methodology of qualitative and quantitative research methods within a study could offer a more holistic comprehension of the research inquiries and future work could benefit from incorporating such mixed methods when designing interventions for SA people with RMD.

## Conclusions

This review has highlighted a series of extrinsic and intrinsic factors that have been identified as barriers or facilitators for SA to engage with EPA. Socioeconomic deprivation that included literacy-related lack of knowledge and disadvantaged backgrounds, adverse environment such as poor weather and safety, lack of social support and sociocultural norms could act as barriers to becoming more physically active in SA communities. Conversely, addressing knowledge gaps, gaining societal support, provision of safe and comfortable environments, culturally acceptable to SA groups, with facilitation to acquire relevant skills regarding EPA-efficacy could promote the engagement of SA with EPA. These findings provide a platform with which to design interventional studies to test whether addressing these factors can result in better engagement with exercise. When presented with opportunity that is culturally acceptable and supported socially and psychologically by society through a client-centred approach, these individuals could develop the skills-mix and exercise efficacy to lead physically active lifestyles.

### Electronic supplementary material

Below is the link to the electronic supplementary material.

Supplementary Material 1

## Data Availability

The datasets used and/or analyzed during the current study are available from the corresponding author on reasonable request.

## Abbreviations

EPA: exercise and physical activity
SA: South Asian
UK: United Kingdom
RMD: rheumatic musculoskeletal disease
SDT: Self-Determination Theory
