# Supplementary material for: Factors facilitating and hindering South Asian immigrant adults from engaging in exercise and physical activity – a qualitative systematic review
Source: BMC Public Health. 2024 May 18;24:1342. doi: 10.1186/s12889-024-18288-1 (PMC11102233; doi:10.1186/s12889-024-18288-1)
Supplement: Supplementary file 1 — Supplementary Material 1 [file 12889_2024_18288_MOESM1_ESM.docx]

Appendix

| **Main search terms used** |
| --- |
| **1. Physical activity (exp)**  **OR**  **2. Exercise (exp)**  **AND**  **3a. South Asian (exp)**  **b. Pakistani (mp) OR**  **c. Bangladeshi (mp) OR**  **d. Indian (mp) OR**  **e. Sikh (mp) OR**  **f. Sri Lankan (mp) OR**  **g. Nepalese (mp) OR**  **h. Bhutanese (mp) OR**  **i. Maldivian (mp)**    **AND**  **4a. Barrier$ (exp) OR**  **b. Challeng$ (mp) OR**  **c. Cultur$ (mp) OR**  **d. Religio$ (mp) OR**  **e. Ethnic$ (mp)**  **OR**  **5a. Enabl$ (exp) OR**  **b. Facilitat$ (mp) OR** |
